# Ustekinumab affects myofibroblast metabolism to alleviate intestinal fibrosis by targeting KDELC1 in Crohn’s disease through multi-machine learning combined with single-cell sequencing analysis

**DOI:** 10.3389/fmed.2024.1476592

**Published:** 2024-10-22

**Authors:** Su Ma, Yongming Kang, Zhonglin Yang, Xingyu Ji, Rui Chen, Xiaomei Sun

**Affiliations:** ^1^Department of Gastroenterology, The First Affiliated Hospital of Jiamusi University, Jiamusi, China; ^2^Department of Gastroenterology, Heilongjiang Provincial Hospital, Harbin, China; ^3^Department of Gastroenterology, Donghai County Hospital, Lianyungang, Jiangsu Province, China

**Keywords:** single-cell RNA sequencing, Crohn’s disease, bioinformatics, machine learning, myofibroblast, Ustekinumab

## Abstract

**Background:**

Ustekinumab (UST), a biologic against interleukin (IL)-12/23, is commonly used to treat Crohn’s disease (CD). Myofibroblast (MF) is known as one of the most important factors causing intestinal fibrosis, and UST has been reported to alleviate this condition. However, the genetic mechanisms underlying UST’s effects on CD remain unclear. This study uses bioinformatics tools to analyze the genes and potential pathways affected by UST in CD, with a focus on its anti-fibrosis effects, providing insights into new therapeutic targets.

**Methods:**

The data downloaded from the Gene Expression Omnibus (GEO) database were analyzed to screen for differentially expressed genes (DEGs). Various machine learning strategies, including the least absolute shrinkage and selection operator (LASSO), support vector machine (SVM), and random forest (RF), were employed to screen for key genes among the DEGs. Functional and pathway enrichment analyses were conducted, and key genes associated with myofibroblast (MF) activity were screened. Finally, endoscopic surgical specimens from CD patients and healthy participants were collected to assess the expression levels of collagen and key genes in intestinal tissues using hematoxylin–eosin (H&E), Masson staining, and immunohistochemistry.

**Results:**

A total of 1,341 DEGs associated with CD were identified. Among them, 738 genes showed low expression in healthy populations but high expression in patients with CD, reduced expression after the treatment of UST. In contrast, 603 genes exhibited high expression in healthy individuals, showed low expression in CD patients, and increased expression after UST treatment. Functional and pathway analysis showed that DEGs were mainly concentrated in response to foreign biological stimuli and bacterial-derived molecules. DEGs are mainly enriched in chemokines, TNF, IL-17, and other signaling pathways. Seven key genes were identified: NCRNA00236, LOC730101, ORP3, XG, UBFD1, KDELC1, and RBP7. Single-cell analysis revealed that KDELC1 was closely related to MF activity. MFs with high KDELC1 expression were significantly enriched in biological functions, signaling pathways, and metabolic processes that promote fibrosis. The experiment showed that UST treatment helped maintain the integrity of intestinal tissue structure, reducing the expression levels of collagen I, KDELC1, and the severity of intestinal fibrosis. The functional and pathway analysis reiterated that DEGs were largely focused on responses to foreign biological stimuli and bacterial-derived molecules, as well as signaling pathways such as chemokines, TNF, and IL-17. Of the identified genes, KDELC1 showed a particularly strong correlation with MF activity in single-cell analysis (R = 0.33, *p* = 3.2e-07). MFs with high KDELC1 expression were closely linked to pathways promoting fibrosis progression, including TGF-*β*, epithelial-mesenchymal transformation, TNF/NF-κB, and related metabolic pathways such as vitamin B6 and arginine.

**Conclusion:**

KDELC1 plays a key role in regulating multiple biological functions, including signaling pathways related to MF. UST alleviates intestinal fibrosis by targeting KDELC1, thereby influencing intramuscular fat metabolism and intercellular communication.

## 1 Introduction

Crohn’s disease (CD) is a progressive, recurrent, chronic, non-specific inflammatory disease of the intestine that causes severe complications such as intestinal fibrosis and intestinal stenosis, which seriously affect the lives of patients. The incidence of CD is on the rise, while the etiology is unknown (1). CD fibrosis, characterized by hidden progression, leads to difficult diagnosis and timely intervention. It was reported that more than half of the patients develop fibrosis and stenosis within 10 years after diagnosis (2). Conventional surgical intervention still causes recurrence and progression of CD fibrosis (3). Even though the inflammation was effectively inhibited in some cases, the progression of fibrosis cannot be completely terminated (4). Until now, intestinal fibrosis from CD has been challenging in clinical practice, and in-depth research and searching for important targets have been among the important tasks (5).

Among various types of cells, myofibroblast (MF) is considered to play an important role in intestinal fibrosis. Upon various cytokines and chemokines, myofibroblasts are activated and then produce excessive extracellular matrix (ECM); their deposition to tissue causes fibrosis with further permanent scarring and dysfunction (6). On the other hand, therapeutic agents are the main method of treating CD. For example, two-thirds of patients with CD have exhibited a considerable response under the treatment of anti-TNF-*α* (7). Unfortunately, the remaining one-third of patients are still at risk of primary or secondary failure and display resistance to anti-TNF-*α* (8). By contrast, Ustekinumab (UST) is a monoclonal antibody that has been used to treat CD with optimal safety, efficacy, and anti-antibody production. However, UST studies on intestinal fibrosis are highly required. Interestingly, it was reported that two patients with CD experienced partial remission of intestinal stenosis after long-term UST maintenance therapy, allowing endoscopies to pass through areas previously inaccessible due to stenosis (9).

To explore the mechanism of UST to alleviate intestinal fibrosis, we analyzed changes in relevant genes and pathways, as well as possible intercellular communication, before and after treatment in UST-treated CD patients. According to our study, in long-term maintenance therapy, UST down-regulates the expression of some genes and reduces the activity of MF, which experiments have proved to have the ability of anti-fibrosis. Our study also identified key genes that are closely associated with MF. Finally, we explore the influence of key genes on MF function, metabolism, and other intercellular communication. Modulating the function of MF provides a new potential target for regulating the progression of intestinal fibrosis.

## 2 Materials and methods

### 2.1 Bulk data download and processing

We downloaded the GSE112366 datasets from the GEO database.^1^ The data were then corrected in R (4.3.1) using the normalizeBetweenArrays function of the “limma” package.

### 2.2 Enrichment analysis

Enrichment analysis of differential genes was performed using the “GSEABase” package, the “ClusterProfiler” package, and the “org.Hs.eg.db” package. The database used for the enrichment analysis was derived from the Gene Ontology.^2^ Use the EnrichGO function for enrichment. If *p* < 0.05, the pathway was considered to be significantly enriched. “ggplot2” package, “ggpubr” package for visualization.

### 2.3 Machine learning

In our results, a total of three machine learning algorithms were used: the Least Absolute Shrinkage and Selection Operator (LASSO), Support Vector Machine (SVM), and Random Forest (RF). For LASSO analysis, we used the “glmnet” package, applying a penalty parameter estimated through 10-fold cross-validation to prevent overfitting during modeling. The “e1071,” “kernlab,” and “caret” packages, along with the rfe function, were utilized for SVM analysis, with the minimum cross-error set as the gene selection criterion. The “randomForest” package was used for RF analysis. We constructed a random forest tree using the randomForest function, with the ntree parameter set to 500, and used the importance function to assess the importance of each gene, selecting the five genes with the highest importance scores as candidate genes.

### 2.4 Acquisition and pre-processing of single-cell transcriptome data

Single-cell transcriptome data were obtained from the GEO database (GEO registration number: GSE134809; see Footnote 1). Quality control was performed in the R environment using standard single-cell processing procedures. The count matrix was read using the Read10X function from the Seurat package (Version 4.0.4), and the latter was further converted to dgCMatrix format. The merge function was used to integrate all individual objects into an aggregate object, and the RenameCells function was used to ensure that all cell labels were unique. We filtered low-quality cells with the following filtering criteria: when a gene was expressed in less than three cells, the gene was deleted. The cell is deleted when the number of genes expressed in a cell is less than 200 or more than 8,000. A global-scaling normalization method, “LogNormalize,” was employed to ensure that the total gene expression in each cell was equal, and the scale factor was set to 10,000. The top 2000 variably expressed genes were returned for downstream analysis using the FindVariableFeatures function. The ScaleData function, “vars.to.regress” option UMI, and percent mitochondrial content were used to regress out unwanted sources of variation. Principal component analysis (PCA) incorporating highly variable features reduced the dimensionality of this dataset, and the first 30 PCs were identified for analysis. The harmony method was used to remove batch effects between samples (10). Cells were down-dimensioned using the UMAP method. Scrublet’s method was used to detect potential doublets.

Clustering analysis was conducted based on the edge weights between any two cells, producing a shared nearest-neighbor graph using the Louvain algorithm, which was implanted through the *FindNeighbors* and *FindClusters* functions. The resolution parameter in the *FindClusters* function was systematically tested between 0.1 and 1. The *clustree* function was used to visualize clustering trees at various resolutions, and it was found that a resolution of 0.5 provided the clearest clustering results. To identify differentially expressed markers for the resulting clusters, the *FindAllMarkers* function was applied, utilizing the default nonparametric Wilcoxon rank sum test with Bonferroni correction. Cell clusters were then annotated based on cell surface markers and gene expression profiles referenced from relevant literature and the cell taxonomy database.^3^

### 2.5 Single-cell differential analysis

We employed the advanced Libra package to conduct differential expression analysis to mitigate the bias introduced by the sparsity of single-cell sequencing data in differential expression analysis. Libra is an R package designed for the analysis of single-cell RNA sequencing (scRNA-seq) data, offering rich functionality for differential expression analysis, cell subpopulation analysis, and other tasks related to single-cell data analysis.^4^

### 2.6 Cellular communication network

Cell–cell interaction analysis was performed using the CellChat (v1.0.0) R package. The ligand and receptor genes expressed by each cell were projected into a manually selected reference communication network, and the probability of communication in each pathway was inferred by gene expression.

### 2.7 Clinical sample collection

A total of 20 patients diagnosed with moderate to severe CD and treated with UST in Heilongjiang Provincial Hospital from October 2021 to October 2023 were collected, and an additional 10 healthy people were recruited as healthy controls. All of whom signed informed consent. Colonoscopy biopsy specimens of CD patients before UST treatment and at 1 year of UST maintenance treatment were taken from the intestinal fibrosis stenosis for follow-up tests. Normal samples come from colonoscopy biopsies of healthy people. The study was conducted strictly in accordance with the principles of the Declaration of Helsinki and was approved by the Ethics Committee of Heilongjiang Hospital (Heilongjiang, China).

The biopsy procedures were conducted according to the previous report (11). All samples were collected from CD patients and healthy controls, with the diagnosis of CD confirmed via endoscopy. The diagnosis of CD was based on a combination of clinical assessments and diagnostic investigations. To determine the position for the collection of samples, the simple endoscopic score for Crohn’s disease (SES-CD) was employed during colonoscopy for all CD patients, classifying them as aCD (active CD, SES-CD ≥ 3) or qCD (quiescent CD, SES-CD ≤ 2). All colonoscopies were conducted according to standard clinical practice for CD diagnosis. The inclusion criteria for patients required diagnosed a confirmed diagnosis of CD based on the European Crohn’s and Colitis Organization criteria. The exclusion criteria for CD patients included patients who had received immunosuppressive therapy for conditions other than inflammatory bowel disease (IBD), those with immune-mediated disease unrelated to IBD, patients with active infections or neoplasms at the time of colonoscopy, and women who were pregnant or lactating during the procedure.

### 2.8 Hematoxylin and Eosin, Masson, and immunohistochemical analysis

Hematoxylin and Eosin (H&E) staining of clinical intestinal tissue samples: Paraffin sections were dewaxed by sequential immersion in xylene for 10 min, followed by another xylene immersion for 5 min. The sections were then treated with anhydrous ethanol for 30 s, followed by 95% alcohol for 30 s, 85% alcohol for 30 s, and 75% alcohol for 30 s. Subsequently, the paraffin sections were immersed in a hematoxylin dye solution for 5 min, rinsed with tap water, and differentiated with 1% hydrochloric acid alcohol for a few seconds. After rinsing with distilled water, the sections were treated with a lithium carbonate-saturated aqueous solution for a few seconds to return to blue, followed by rinsing with running water. The sections were then immersed in eosin dye solution for 30 s, followed by a final rinse with running water. The dehydration process involved sequential immersion in 75% alcohol for 30 s, and 95% alcohol for 30 s, followed by two rounds of anhydrous ethanol for 30 s each. Finally, the sections were cleared in xylene I for 30 s, xylene II for 30 s, and sealed with neutral gum.

Masson’s trichrome staining for intestinal tissue specimens follows these steps:

1. Stain with Weigert’s solution (a mixture of equal parts of A and B solutions) for 5 min, followed by a gentle rinse under running water.
2. Differentiate the stain by briefly treating it with 1% hydrochloric acid alcohol for a few seconds, then wash the specimen for several minutes with water.
3. Apply Lichun red acid fuchsin dye for 5 min, and rinse lightly with running water afterward.
4. Treat the specimen with a phosphomolybdate solution for 5 min, then pour off the solution.
5. Re-stain using an aniline blue dye solution for 3 min, then pour off the dye.
6. Wash the specimen with 1% glacial acetic acid solution (made by adding 1 mL glacial acetic acid to 99 mL distilled water) until the blue coloration no longer runs off the sections.
7. Lightly rinse with 95% alcohol, dehydrate the specimen with anhydrous ethanol, and clear with xylene.
8. Finally, seal the slide using neutral gum.

Immunohistochemical staining of intestinal tissue specimen: The dewaxed slices were immersed in 3% hydrogen peroxide at room temperature for 25 min, protected from light, to block endogenous peroxidase activity. For collagen I/KDELC1 staining, antigen retrieval was performed using EDTA (pH 8.0) under high temperature and pressure, steaming the sections for 3 min. After cooling to room temperature, the sections were rinsed three times with PBS for 5 min each.

The sections were then blocked with goat serum and incubated at room temperature for 60 min, away from light. After gently shaking off the blocking solution, the appropriate dilution of the primary antibody in PBS was applied to the tissue sections, which were then placed flat in a humidified chamber at 4°C for overnight incubation (with a small amount of water in the chamber to prevent antibody evaporation).

The slides were washed three times with PBS (pH 7.4) for 5 min each. After slight drying, the secondary antibody (corresponding to the species of the primary antibody) was added and incubated at room temperature for 60 min in the dark. The slides were again washed three times in PBS (pH 7.4) for 5 min each.

Following drying, the sections were treated with freshly prepared DAB solution, and the color development was monitored under a microscope. Positive staining appeared as brown or yellow. The color development was stopped by rinsing the sections with tap water.

The sections were counterstained with hematoxylin for 5–10 min, rinsed with tap water, differentiated in 1% hydrochloric acid alcohol for a few seconds, and then rinsed again with tap water. A lithium carbonate-saturated aqueous solution was applied to return the sections to a blue color for 1 min, followed by a final rinse with tap water.

The paraffin sections were sequentially dehydrated in 75% alcohol for 30 s, 95% alcohol for 30 s, and twice in absolute ethanol for 30 s each. The sections were then cleared in xylene I and xylene II for 30 s each and mounted with neutral gum.

Three 200 × high-magnification areas were randomly observed for each indicator, and the average integrated optical density per stained area (IOD/area) value was calculated using Image Pro Plus software.

### 2.9 Statistical analysis

SPSS 29.0 statistical software was used to analyze the data. The t-test analysis method was used to analyze the differences among sample groups. The one-way ANOVA method was used to analyze the expression of key genes in each sample group. Spearman analysis was used to calculate the correlation analysis, and Graphpad Prism 10.1.2 was used to plot. *p* < 0.05 was considered statistically significant (*p < 0.05, **p < 0.01, ***p < 0.001, ****p < 0.0001).

## 3 Results

### 3.1 Baseline data characteristics

As shown in Table 1, the sample for the study is from a total of 20 patients with moderate to severe CD, including 15 men and five women, with an average course of disease of 5.70 ± 3.28 years and an average age of 32.45 ± 12.37 years. A total of nine patients were afflicted with severe CD and 11 patients with moderate CD. 11 patients (55%) had a history of smoking and drinking. The Montreal classification included 16 patients with stage A2 (80%), four patients with stage A3 (20%), seven patients with B2 + L1 (35%), six patients with B2 + L2 (30%), and seven patients with B2 + L3 (35%). According to the enrollment criteria, all patients with intestinal stenosis were selected.

**Table 1:** Clinical features of CD patients before treatment initiation.

	Age (years)	Gender (Men/ Women)	Montreal classification	Medical history (years)	Simplified CDAI (score)	Smoking and drinking	Biopsy site
Patient 1	32	M	A2B2L1	11	11	N	Terminal ileum
Patient 2	41	M	A3B2L1L2	7	17	Y	Colon ascendens
Patient 3	34	M	A2B2L1	7	15	Y	Terminal ileum
Patient 4	36	F	A2B2L1p	9	14	Y	Terminal ileum
Patient 5	49	M	A3B2L1p	11	15	Y	Terminal ileum
Patient 6	55	M	A3B2L3p	6	18	Y	Ileum
Patient 7	27	M	A2B2L3p	2	17	N	Ileum
Patient 8	34	M	A2B2L3p	2	19	Y	Ileum
Patient 9	17	M	A2B2L2	3	16	N	Colon ascendens
Patient 10	34	F	A2B2L3p	4	18	N	Ileum
Patient 11	28	M	A2B2L2	7	19	Y	Colon ascendens
Patient 12	32	F	A2B2L2p	12	15	Y	Colon ascendens
Patient 13	17	M	A2B2L2	4	13	N	Colon ascendens
Patient 14	25	F	A2B2L3p	4	20	N	Ileum
Patient 15	32	M	A2B2L2p	8	20	Y	Colon ascendens
Patient 16	62	M	A3B2L1L2	2	14	Y	Terminal ileum
Patient 17	37	M	A2B2L3	3	12	Y	Ileum
Patient 18	17	M	A2B2L1L2	2	14	N	Terminal ileum
Patient 19	22	F	A2B2L2	7	20	N	Colon ascendens
Patient 20	18	M	A2B2L3	3	11	N	Ileum

In the case of the abdominal symptoms, all patients suffered from abdominal pain and diarrhea; 7 patients (35%) had abdominal mass, and 18 patients (90%) had bloody stools. Among the systemic symptoms, all patients had fatigue and weight loss, and 12 patients (60%) had fever. In terms of parenteral manifestations, eight patients (40%) had oral ulcers, one patient (5%) had spinal arthritis, and eight patients (40%) had peripheral arthritis. Prior to UST treatment, nine patients (45%) were treated with one biologic agent, all of which were anti-TNF-*α* agents: Infliximab. In addition, 11 patients (55%) took other drugs, including mesalazine and glucocorticoid drugs. Among the complications, five patients (25%) had intestinal obstruction, nine patients (45%) had perianal lesions, five patients (25%) had anal fistula, and one patient (5%) had intestinal fistula (Table 2).

**Table 2:** Clinical symptoms, parenteral manifestations, complications, and other information of CD patients before treatment initiation.

Symptom	CD patients prior to initiation of treatment (*n* = 20)
Abdominal symptoms	
Abdominal pain	20 (100%)
Diarrhea	20 (100%)
Abdominal mass	7 (35%)
Bloody stool.	18 (90%)
Constitutional symptoms	
Fever	12 (60%)
Loss of weight	20 (100%)
Fatigue	20 (100%)
Extra-intestinal manifestations	
Dental ulcer	8 (40%)
Spondylarthritis	1 (5%)
Peripheral arthritis	8 (40%)
Drug application information	
Apply a single biological agent	9 (45%)
In combination with other medications	11 (55%)
Complication	
Intestinal obstruction	5 (25%)
Perianal disease	9 (45%)
Anal fistula	5 (25%)
Intestinal fistula	1 (5%)

### 3.2 The difference in fibrin deposition, collagen І, and TGF-*β*1 expression in intestinal stenosis tissues of CD patients upon the treatment of UST

**Figure 1:**
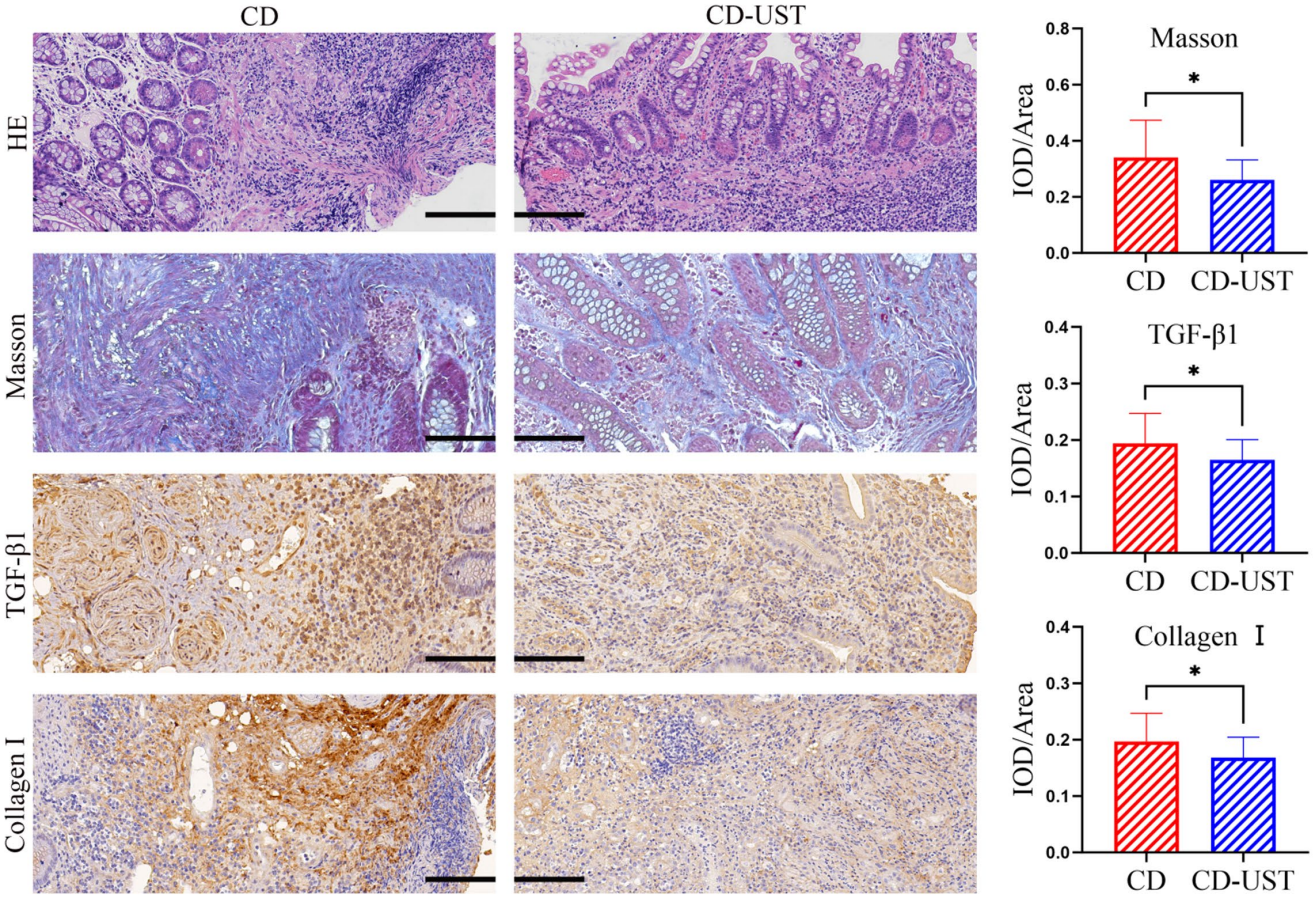
Comparison of intestinal stenosis morphology and collagen I, TGF-β1, and fibrosis degree in CD patients before treatment and 1 year after maintenance treatment (Visual field multiple: 200 times, *p* < 0.05 considered statistically significant, *p < 0.05).

A histology analysis using H&E and Masson’s trichrome stain was conducted to study the impact of UST treatment on intestinal stenosis tissues. Immunohistochemical staining analysis of the expression of collagen I and TGF-β 1 in intestinal stenosis upon the treatment of UST. As shown in Figure 1, the sample of one patient exhibited incomplete colon tissue structure and disordered arrangement of cells before UST treatment, as well as obvious epithelial damage and inflammatory cell infiltration. In contrast, the colon structure and organization were relatively complete after the UST treatment. The mucosal surface was relatively flat. The epithelial cells were arranged in orderly and obvious inflammatory infiltration. The above results indicated that treatment of UST maintained CD patients’ colonic tissue structure and epithelial integrity.

As shown in Figure 1 and Table 3, the histology analysis with Masson staining showed that a small amount of fibrin existed between the submucosa and lamina propria in the narrow intestine after treatment. By contrast, a wide distribution of fibrin was exhibited in the narrow intestine before treatment. Moreover, immunohistochemistry showed that in the narrow intestinal tissues before treatment, collagen І was widely expressed in the submucosa and lamina propria tissues, and its distribution was denser than that in the intestinal tissues after treatment.

**Table 3:** Comparison of average optical density of collagen I, TGF-β1 and Masson stain in intestinal tissues before and after treatment (x ± s).

	n	CD	CD-UST	*p*-value
Collagen I	20	0.20 ± 0.05	0.17 ± 0.04	*p* < 0.05
Masson	20	0.34 ± 0.13	0.26 ± 0.07	*p* < 0.05
TGF-β1	20	0.19 ± 0.05	0.16 ± 0.04	*p* < 0.05

### 3.3 The difference in MF count of the CD stenotic intestine before and after treatment

**Figure 2:**
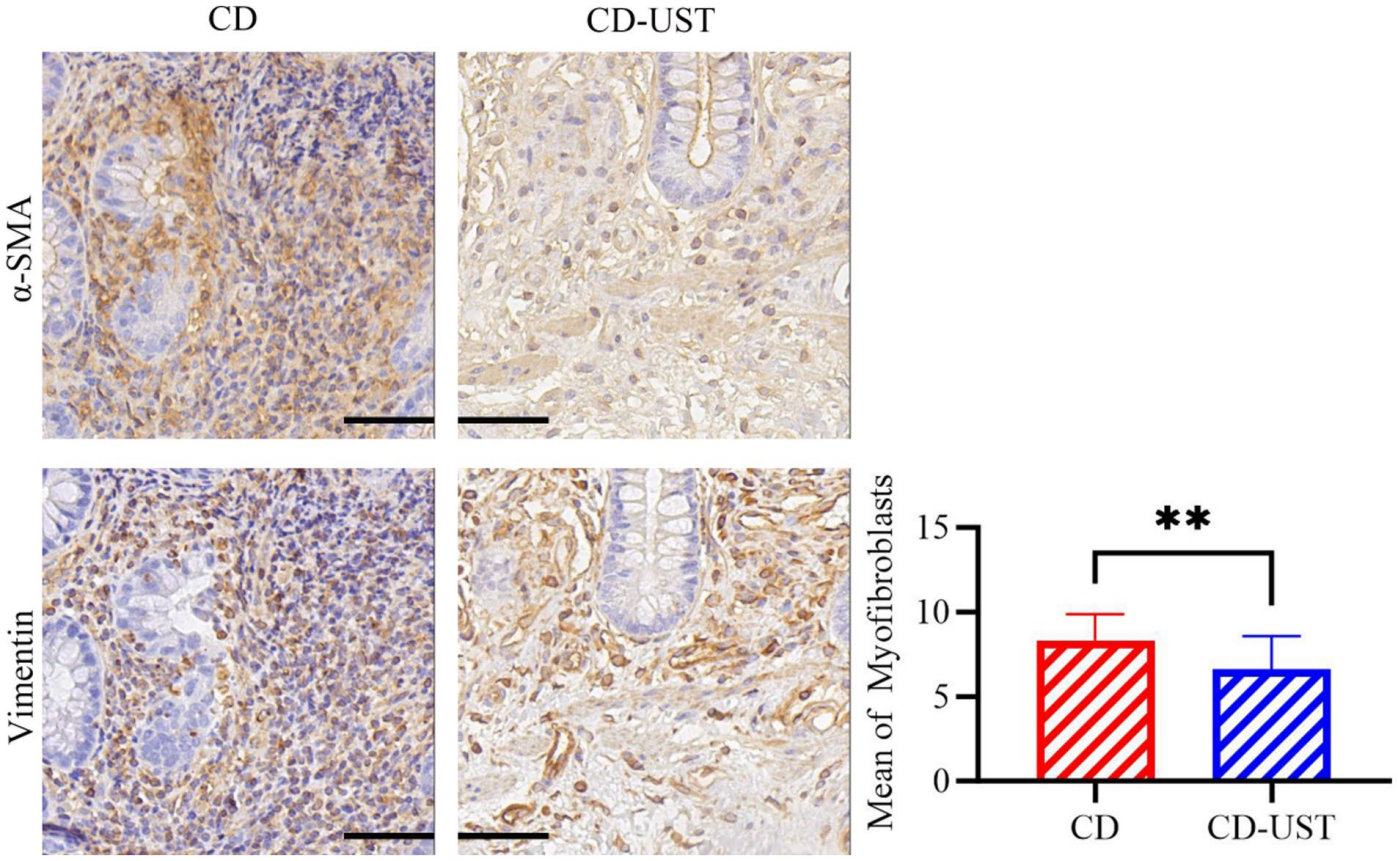
Average number of MF in *α* -SMA and Vimentin double-positive regions of the serial intestinal sections before and after treatment of CD patients (Field multiple: 400 times, a *p*-value of<0.05 was considered statistically significant, *p < 0.05, *p < 0.01).

Furthermore, the immunohistochemical staining was carried out to identify the type of cells in intestinal tissue that simultaneously express *α*-SMA and vimentin. Continuous section staining was performed to identify MF co-expressing α-SMA and vimentin. Then, the relationship between the number of double-positive cells in the intestinal tissue of CD patients before and after UST treatment and the treatment time was calculated and analyzed. The data in Figure 2 and Table 4 indicate a statistically significant decrease in MF number shown by immunohistochemistry at 1 year of long-term maintenance therapy with UST.

**Table 4:** Comparison of MF counts for α -SMA and Vimentin in CD and CD-UST (x ± s).

	n	CD	CD-UST	*p*-value
MF	20	8.32 ± 1.57	6.64 ± 1.94	*p* < 0.01

### 3.4 UST treatment affects associated genes and pathways in CD

**Figure 3:**
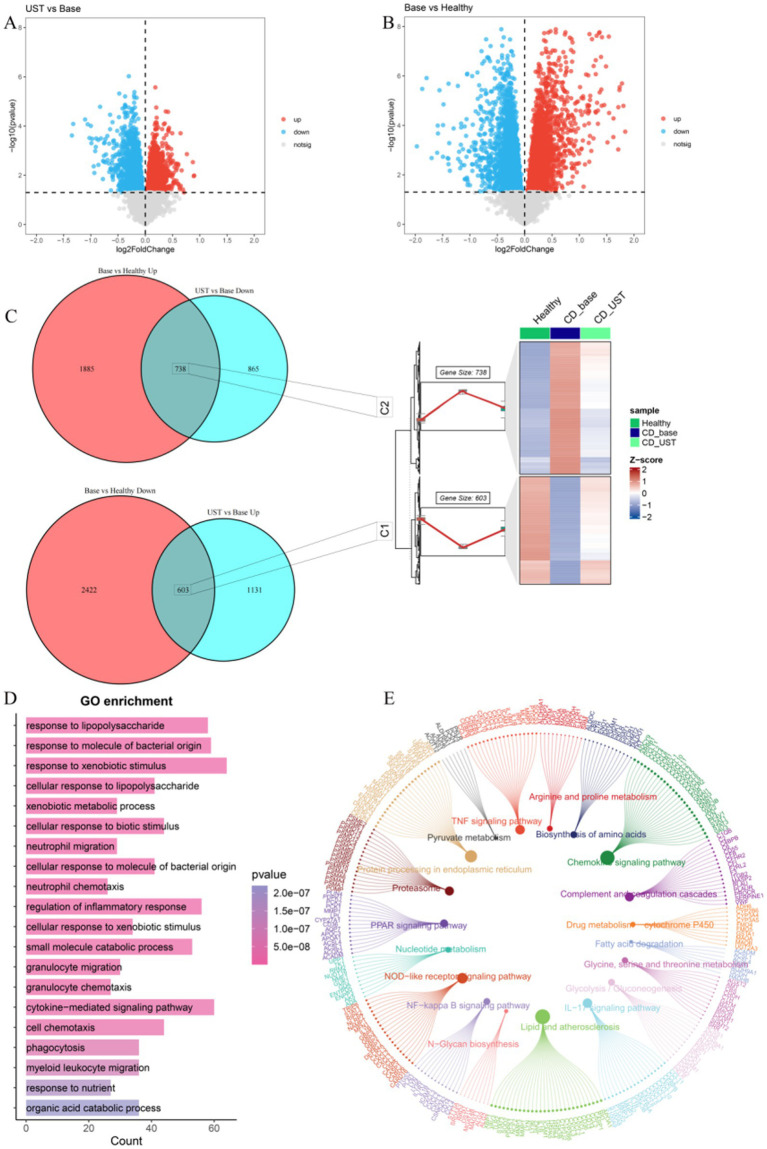
UST influences related genes and pathways in CD. (A) Volcanic maps for post-treatment and baseline difference analysis of UST. (B) Volcano maps for healthy control groups and baseline difference analyses. (C) 603 DEGs with high expression of C1 in a healthy control group, low expression in CD, and increased expression after UST treatment. The expression of C2 was low in a healthy control group, high in CD, and decreased in 738 DEGs after UST treatment. (D) GO enrichment analysis results. (E) Results of KEGG enrichment analysis.

In the GSE112366 dataset, we used untreated CD patients as the baseline. By analyzing the differences between UST treatment and baseline, 1,734 upregulated DEGs and 1,603 downregulated DEGs were identified (Figure 3A). By comparing the differences between UST treatment and healthy people, 2,623 upregulated DEGs and 3,025 upregulated DEGs were identified (Figure 3B). The expression of 738 DEGs in C2 was low in healthy people, high in CD, and decreased after UST treatment. The 603 DEGs in C1 were highly expressed in healthy people, low in CD, and increased after UST treatment (Figure 3C). Subsequently, we performed functional enrichment analyses to predict the biological functions of 1,341 DEGs. The genes that exhibit high expression in the healthy group, low expression in CD, and increased expression after UST treatment are named the C1 group, and the genes that display low expression in the healthy group, high expression in CD, and increased expression after UST treatment are named the C2 group. The results of GO analysis showed that it was mainly enriched in response to foreign biological stimuli, cytokine-mediated signaling pathways and responses to molecules of bacterial origin, lipopolysaccharide responses, and regulation of inflammatory responses (Figure 3D). KEGG pathway analysis showed that there was an enrichment in the various pathways, including lipid and atherosclerosis, chemokine signaling pathway, endoplasmic reticulum protein processing, NOD-like receptor signaling pathway, TNF signaling pathway, and IL-17 pathway (Figure 3E).

### 3.5 Screen key downstream targets of UST through machine learning

**Figure 4:**
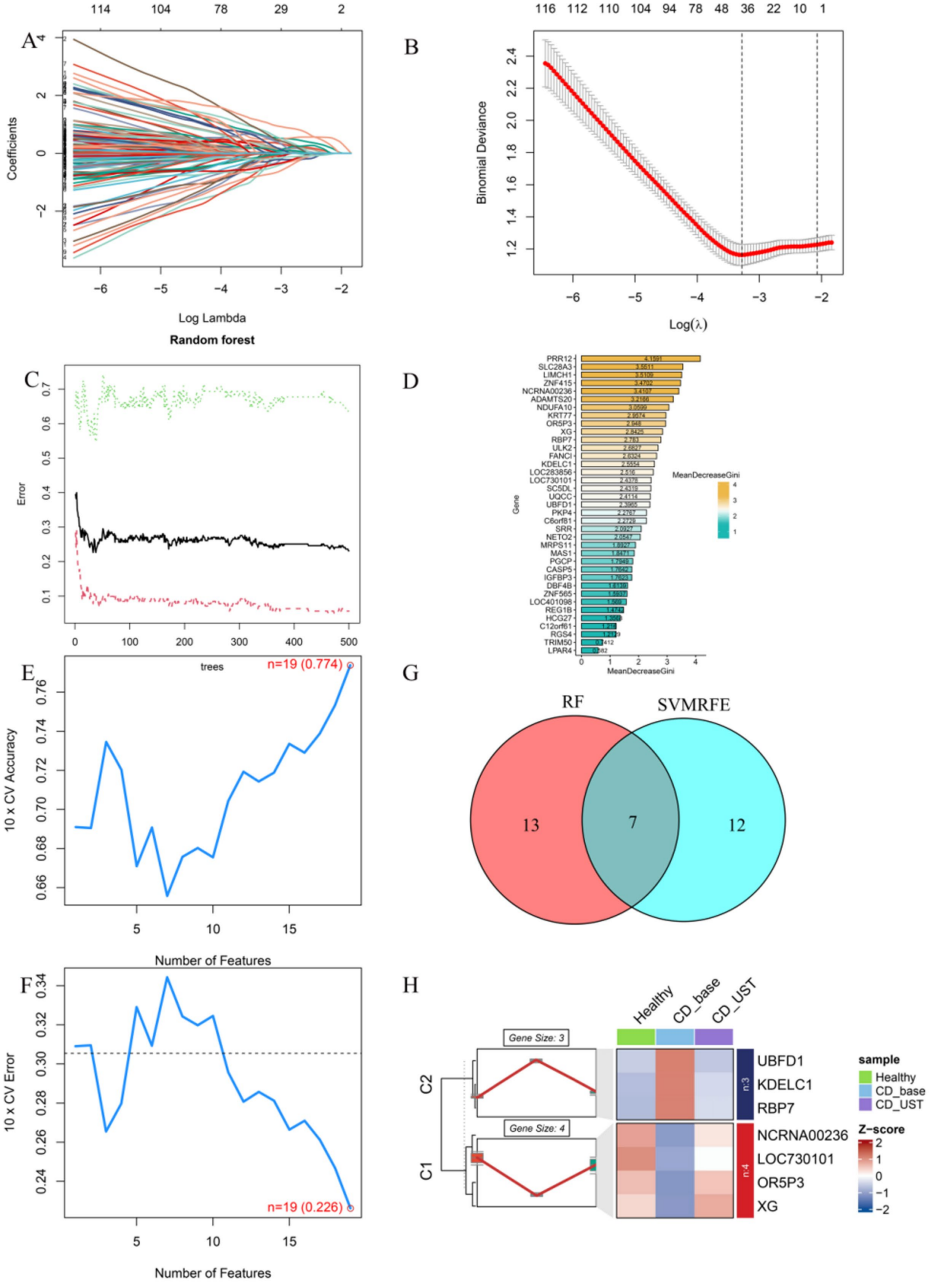
Screen key downstream targets of UST through machine learning. (A,B) The Lasso regression model was used to screen candidate genes, and 37 genes corresponding to the lowest point of the regression curve were the most suitable candidate genes. (C,D) error shown by the random survival forest algorithm, these genes were sequenced according to the MeanDecreaseGini value. (E,F) The SVM algorithm shows the points with the highest accuracy and lowest error rate. (G) Venn diagram showing seven common genes between LASSO and RF algorithms, which were identified as key hub genes. (H) C1 was a gene with high expression in the healthy group, low expression in CD, and increased expression after UST treatment (NCRNA00236, LOC730101, ORP3, XG). C2 is a gene with low expression in the healthy group, high expression in CD, and decreased expression after UST treatment (UBFD1, KDELC1, RBP7).

LASSO results showed that a total of 37 genes were screened (Figures 4A,B). Random forest models scored and sequenced these candidate genes (Figures 4C,D). The SVM algorithm shows that *n* = 19 is the point with the highest accuracy (0.774) and the lowest error rate (0.226; Figures 4E,F). The results of RF and SVM were intersected to obtain seven genes (Figure 4G). The results showed that three genes (UBFD1, KDELC1, and RBP7) were underexpressed in the healthy group and overexpressed in the CD group, and their expression levels decreased after UST treatment. Four genes (NCRNA00236, LOC730101, ORP3, and XG) were highly expressed in the healthy group and lowly expressed in CD, and their expression levels increased after UST treatment (Figure 4H).

### 3.6 Single-cell analysis determined that up-regulation of KDELC1 promoted infiltration of MF

**Figure 5:**
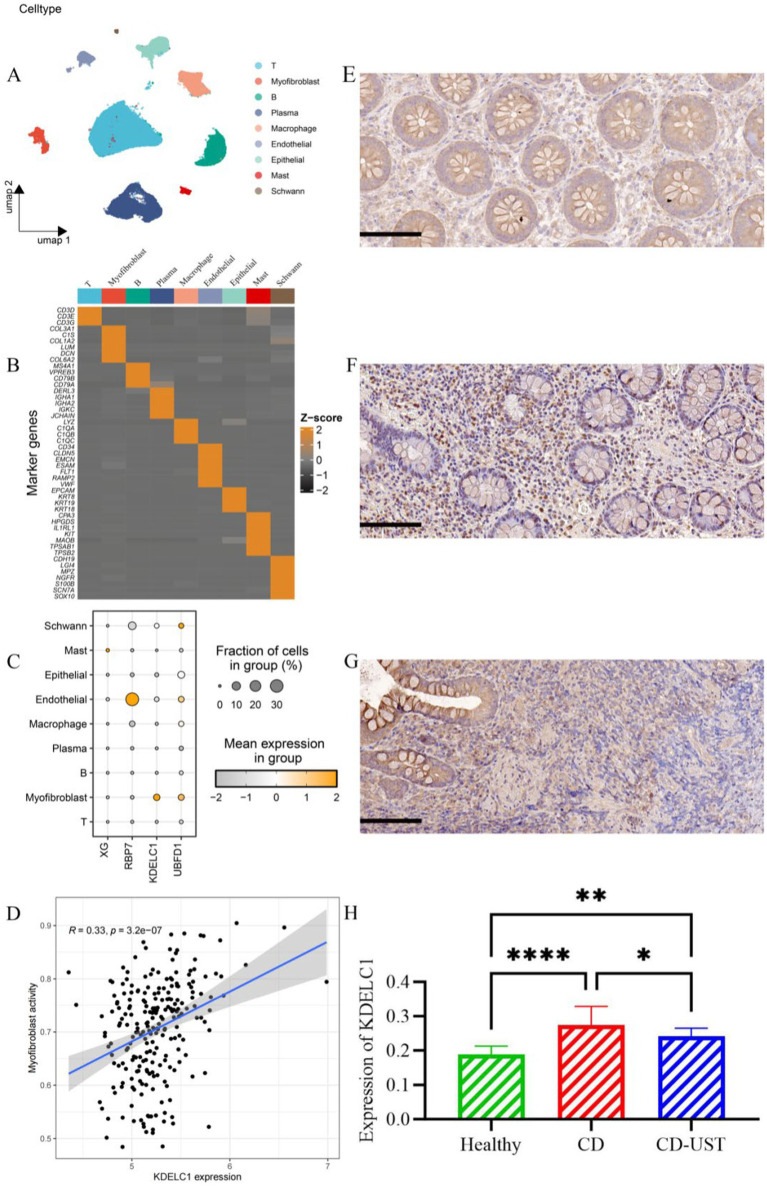
Cell types, correlation analysis, and expression of KDELC1 in CD. (A) Umap shows different cell types in the gut of CD. (B) Heat maps of marker gene expression levels in 9 cell types. (C) Bubble map showing the expression of top1 marker genes in nine cell types. (D) Correlation between MF activity and KDELC1 in CD. (E-H) Expression of KDELC1 in the healthy control group, CD patients before treatment, and CD patients after treatment with UST. Visual field multiple: 200, *p* < 0.05 considered statistically significant (*p < 0.05, * *p < 0.01, ***p < 0.001, ****p < 0.0001).

Single-cell analysis revealed that a total of 9 cell types (T cell, MF, B cell, plasma cell, macrophage, endothelial cell, epithelial cell, Mast cell, Schwann cell) have been annotated (Figures 5A,B). Notably, UBFD1 is highest expressed in MF cells, Schwann cells, and endothelial cells, RBP7 was significantly expressed in endothelial cells, and XG is highly expressed in mast cells (Figure 5C). Through correlation analysis, it was found that the increase in KDELC1 expression level was positively correlated with MF activity (R = 0.33, *p* = 3.2e-7) (Figure 5D). Immunohistochemistry analysis showed that KDELC1 was lowly expressed in the intestinal tissues of healthy subjects, highly expressed in the intestinal tissues of untreated CD patients and decreased in the intestinal tissues of CD patients treated with UST. The comparison between the two groups was statistically significant (Figures 5E–H). Therefore, UST treatment significantly reduced the expression of KDELC1 in the intestinal tissues of CD, but it did not reach the level of healthy subjects (Table 5).

**Table 5:** Expression of KDELC1 in the healthy control group, CD patients, and CD patients after UST treatment (x ± s).

	Health	CD	CD-UST
The expression of KDELC1	0.19 ± 0.01	0.27 ± 0.06	0.24 ± 0.04

### 3.7 Influence of KDELC1 expression on MF function

**Figure 6:**
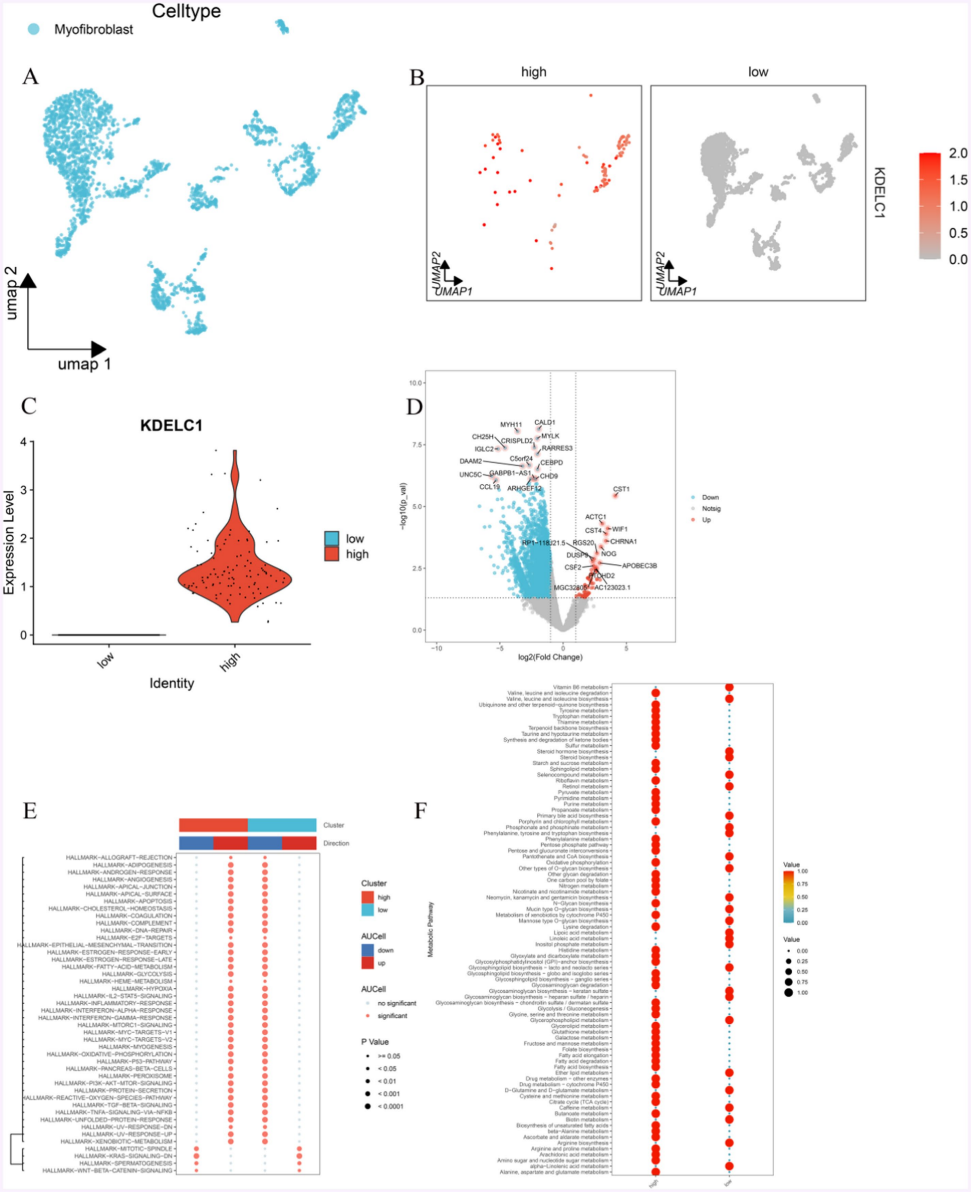
Effect of KDELC1 expression on MF function. (A) UMAP shows MF. (B,C) MF was divided into a high-expression group and a low-expression group according to the expression level of KDELC1. (D) By analyzing the difference between the two groups of MF, we obtained the gene volcano map of differential expression. (E) GSVA analysis of the Hallmark channel between MF with high expression of KDELC1 and MF with low expression of KDELC1. (F) Metabolic analysis of MF with high expression of KDELC1 and MF with low expression of KDELC1.

MF was divided into two groups according to the expression level of KDELC1: MF with high expression of KDELC1 and MF with low expression of KDELC1 (Figures 6A–C, 4B,C). In addition, the genes related to these two groups of cells were analyzed to make a volcano map, and the differentially expressed genes were obtained (Figure 6D).

In this volcanic map, we can observe that the genes that are significantly upregulated are: CST1, ACTC1, CST4, WIF1, CHRNA1, NOG, RGS20, RP1-118 J21.5, DUSP9, APOBEC3B, CSF2, PTCHD2, MGC32805, AC123023.1; Significantly down-regulated genes were CALD1, MYH11, MYLK, CRISPLD2, CH25H, IGLC2, RARRES3, C5ORF24, DAAM2, CEBPD, UNC5C, GABPB1-AS1, CHD9, CCL19, ARHGEF12 (Figure 6D).

With the analysis of GSEA enrichment, it was found that the MF-promoted pathways with high KDELC1 expression were as follows: TNF/NF-kB, TGF-*β*, PI3K-AKT, P53, oxidative phosphorylation, IL-2/STAT5, epithelial-mesenchymal transformation, inflammatory response, lipogenesis, MTORC1, interferon, and other signaling pathways were inhibited, while WNT/β-Catenin and KRAS signaling pathways were inhibited (Figure 6E). On the contrary, in MF with low expression of KDELC1, TNF/NF-kB, TGF-β, PI3K-AKT, P53, oxidative phosphorylation, IL2/STAT5, epithelial-mesenchymal transformation, inflammatory response, lipogenesis, MTORC1, interferon and other signaling pathways were inhibited as follows: signaling pathways such as WNT/β-Catenin and KRAS are in the inhibited state, while signaling pathways such as WNT/β-Catenin and KRAS are in the promoted state (Figure 6E).

Meanwhile, we also observed the differences in metabolism between both groups of MF. The group of MF with high expression of KDELC1 has lower metabolic activity compared with that with low expression of KDELC1, including linolenic acid metabolism, arginine biosynthesis, biotin metabolism, caffeine metabolism D-glutamine and D-glutamic acid metabolism, ether lipid metabolism, glycerophospholipid metabolism, glycosaminoglycan biosynthesis-heparin sulfate/heparin, glycosaminoglycan biosynthesis-keratin sulfate, glycosaminoglycan biosynthesis-lactate and neolactic acid series, phosphoinositol metabolism, linoleic acid metabolism, lipoic acid metabolism, mannose O-glycan biosynthesis, mucin O-glycan biosynthesis, neomycin, kanamycin and gentamicin biosynthesis, other types of O-glycan biosynthesis, pantothenate and coenzyme A biosynthesis, phenylalanine, tyrosine and tryptophan biosynthesis, phosphate and phosphate biosynthesis Metabolism, primary bile acid biosynthesis, retinol metabolism, selenium compound metabolism, steroid biosynthesis, steroid hormone biosynthesis, and as well as the metabolism of vitamin B6 (Figure 6F).

Those with higher metabolic activity include: alanine, aspartic acid and glutamic acid metabolism, amino sugar and nucleotide sugar metabolism, arachidonic acid metabolism, arginine and proline metabolism, ascorbic acid and aldehyde salt metabolism *β—*alanine metabolism, biosynthesis of unsaturated fatty acids, butyrate metabolism, citric acid cycle (TCA cycle), cysteine and methionine metabolism, drug metabolism, cytochrome P450, drug metabolism, fatty acid biosynthesis, fatty acid degradation, fatty acid elongation, folate biosynthesis, fructose and mannose metabolism, galactose metabolism, glutathione metabolism, glyceride metabolism, metabolism of glycine, serine and threonine, glycolysis/gluconeogenesis, glycosaminoglycan biosynthesis—chondroitin sulfate/chitosan sulfate, glycosaminoglycan degradation, glycosaminoglycan biosynthesis—ganglion series, glycosaminoglycan biosynthesis—Globulin and isoglobulin series, glycosylphosphatidylinositol (GPI)—anchored biosynthesis, glyoxylate and dicarboxylate metabolism, histidine metabolism, lysine degradation The metabolic effects of cytochrome P450 on exogenous drugs N-glycan biosynthesis, metabolism of niacin and nicotinamide, nitrogen metabolism, folate carbon pool, degradation of other polysaccharides, oxidative phosphorylation, mutual conversion of pentose and gluconate, pentose phosphate pathway, phenylalanine metabolism, porphyrin and chlorophyll metabolism, propionic acid metabolism, purine metabolism, pyrimidine metabolism, pyruvate metabolism, riboflavin metabolism, sphingolipids metabolism, starch and sucrose metabolism, sulfur metabolism, ketone synthesis and degradation, taurine and taurine metabolism, terpenoid skeleton biosynthesis, thiamine metabolism, tryptophan metabolism, tyrosine metabolism, ubiquinone and other terpenoid quinone biosynthesis, degradation of valine, leucine, and isoleucine (Figure 6F).

In addition, we can observe that in MF with high expression of KDELC1, the metabolism of vitamin B6 is downregulated, and the biosynthesis activity of various amino acids is low, such as arginine, valine, leucine and isoleucine, phenylalanine, tyrosine, and tryptophan. However, oxidative phosphorylation, the pentose phosphate pathway, and fatty acid metabolism are highly active. The opposite effect was observed in MF with low KDELC1 expression (Figure 6F).

### 3.8 Interactions of MF with other cells

**Figure 7:**
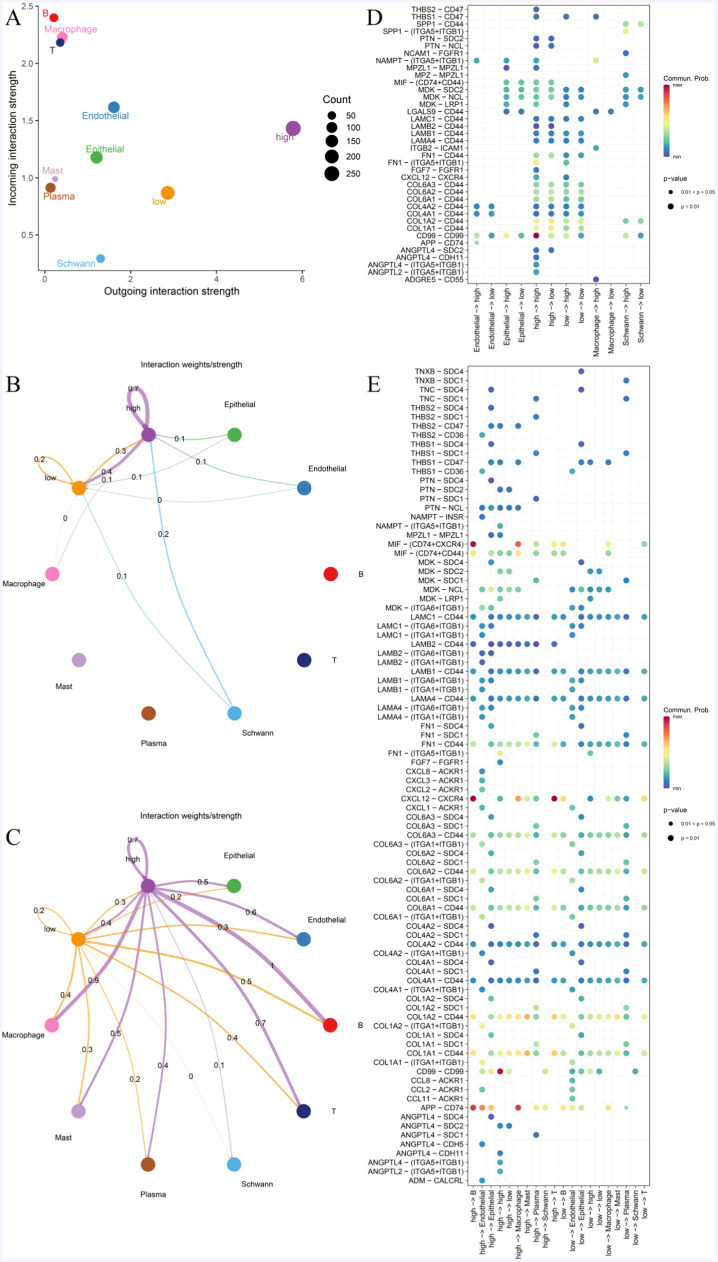
Cell-to-cell communication network. (A) The interaction strength of MF with different expressions of KDELC1 was compared in a two-dimensional graph. (B,C) The intensity of MF interactions with other cells at different KDELC1 expression levels. The colors of bubbles and lines in the figure indicate different cell types, and the larger the number and the thickness of the lines indicate, the greater the weight/intensity of interactions between cells. (D) Bubble plots represent the most significant ligand-receptor interactions between pairs of different cell types, with the size of each bubble representing the p-value and the color representing the average expression of ligand and receptor genes. (E) The bubble map shows receptor and ligand pairs between MF with different KDELC1 expression levels and other cells.

To explore the characteristics of MF with different expressions of KDELC1 levels interacting with other cells, we performed cell communication analyses based on single-cell data and CellChat R packets. MF mainly communicates with macrophages, T cells, B cells, epithelial cells, and endothelial cells (Figure 7A). MF with high expression of KDELC1 will promote communication between other cells in the immune microenvironment, thus causing the disturbance of the immune microenvironment (Figure 7A). When MF acts as incoming, the signal strength of MF itself and between MF and epithelial cells, endothelial cells, macrophages, and Schwann cells can be observed (Figure 7B). The results show that there is a complex communication relationship between different cells (Figures 7B,C). When MF is outgoing, it can be observed that MF with high expression of KDELC1 has higher signal intensity with macrophages, T cells, B cells, epithelial cells, and endothelial cells. In addition, it was clearly observed that MF with high KDELC1 expression had a higher interaction weight with other cells compared to MF with low KDELC1 expression (Figure 7C).

We observed that the CD99-CD99 pathway was most active in MF with high KDELC1 expression and less active in MF with low KDELC1 expression (Figure 7D). We listed that when MF is used as a ligand, it can communicate with a variety of cells through ligand receptors. Compared with MF with low expression of KDELC1, MF with high expression of KDELC1 has more interactions with B cells, macrophages, and endothelial cells through APP-CD74. CXCL12-CXCR4 has a strong interaction with B cells and T cells. There was a higher degree of interaction with B cells and macrophages via MIF- (CD74 + CXCR4; Figure 7E).

## 4 Discussion

Although a large number of studies have been conducted in recent years, the mechanism of intestinal fibrosis is still unclear. In the case of CD patients with fibrostenosis phenotype, endoscopic therapy and surgical treatment are the main modalities. However, the lesions of CD are jumpy with segmental distribution in different parts of the intestine, resulting in the scope of surgery varying from person to person. In addition, too little intestinal retention after surgery may lead to severe short bowel syndrome, and residual inflammation at the incisal margin is significantly correlated with the risk of postoperative recurrence (12). In CD, smooth muscle cells in the intestine can differentiate into MF under the stimulation of pro-fibrotic factors, which leads to excessive deposition of ECM and thickening of the proper muscle layer of the intestine, thus forming intestinal stenosis (13). A recent study revealed that CHMP1A, TBX3, and RNF168 regulate collagen expression of MF in the ileum and colon of CD (14). Therefore, regulating the activity of MF is essential for influencing intestinal fibrosis.

We identified 141 pre-treatment CD samples, 62 post-treatment UST samples, and 26 healthy normal subjects from the GSE112366 dataset. Based on functional and pathway enrichment analysis, we observed that UST affected genes involved in cytokine-mediated signaling pathways, responses to inflammation, and lipopolysaccharides. Cytokines play an important role in the pathogenesis of CD. Some cytokines can promote inflammation, such as TNF-*α*, IL-12, IL-17, IL-23, etc., while some other cytokines can resist inflammation, such as IL-10, etc. In addition, chronic and repeated inflammation will cause some cytokine disorders and then promote intestinal fibrosis, such as IL-11, IL-33, IL-34, and IL-36 (15). Studies have confirmed that compared with healthy people, the intestinal and systemic proinflammatory cytokines are increased in patients with CD, while the anti-inflammatory cytokines are inhibited (16, 17). At present, CD targeting cytokine therapy (including anti-TNF-α, anti-IL-12/23, etc.) has achieved significant efficacy, can alleviate symptoms in the short term, and maintains the ability of mucosal healing in the long term. In addition, these genes also influenced responses to foreign biological stimuli and to molecules of bacterial origin. Lipopolysaccharide (LPS), the cell wall component of gram-negative bacteria, regulates the permeability of intestinal epithelium through toll-like receptor-4, and inhibition of TLR4 expression contributes to alleviating intestinal inflammation (18, 19).

KDELC1, also known as POGLUT2, regulates the Notch signaling pathway by targeting Notch signaling members (NOTCH1 and NOTCH3) (20). Previous reports have shown that Notch signaling is activated in the inflammatory mucosa of IBD patients and contributes to regeneration and proliferation (21). It also mediates the glycosylation of Fibrillin-1(FBN1), Fibrillin-2(FBN2) and latent transforming growth factor *β*-binding protein 1(LTBP1) (22). They are both components of ECM, and FBN1 is the main component of microfibril in ECM, providing support and integrity for a variety of organ tissues (23, 24). In addition, the TGF-β signaling pathway, which is closely related to intestinal fibrosis, can be regulated (25). Our experimental results were consistent with the results of the analysis: KDELC1 was low in the normal intestinal tissues, high in the intestinal tissues of patients with CD, and decreased after anti-IL-12/23 treatment.

At present, single-cell RNA sequencing (scRNA-seq) technology is one of the important methods for analyzing the cellular and molecular composition of CD, providing researchers with a more in-depth understanding of CD opportunities. In addition, single-cell sequencing technology has important advantages for analyzing cell-specific gene expression, cell types, and cell–cell interactions in different tissues (26). For example, an analysis based on single-cell sequencing found that regulating the activity of CDH11 may affect the production of ECM by MF, thereby alleviating intestinal fibrosis (27), which is also consistent with previous results in a mouse model of colitis (28). A recent single-cell technique has identified modules related to resistance to anti-TNF therapy, which will deepen the understanding of CD heterogeneity and provide more targeted assistance in treatment response (29).

**Figure 8:**
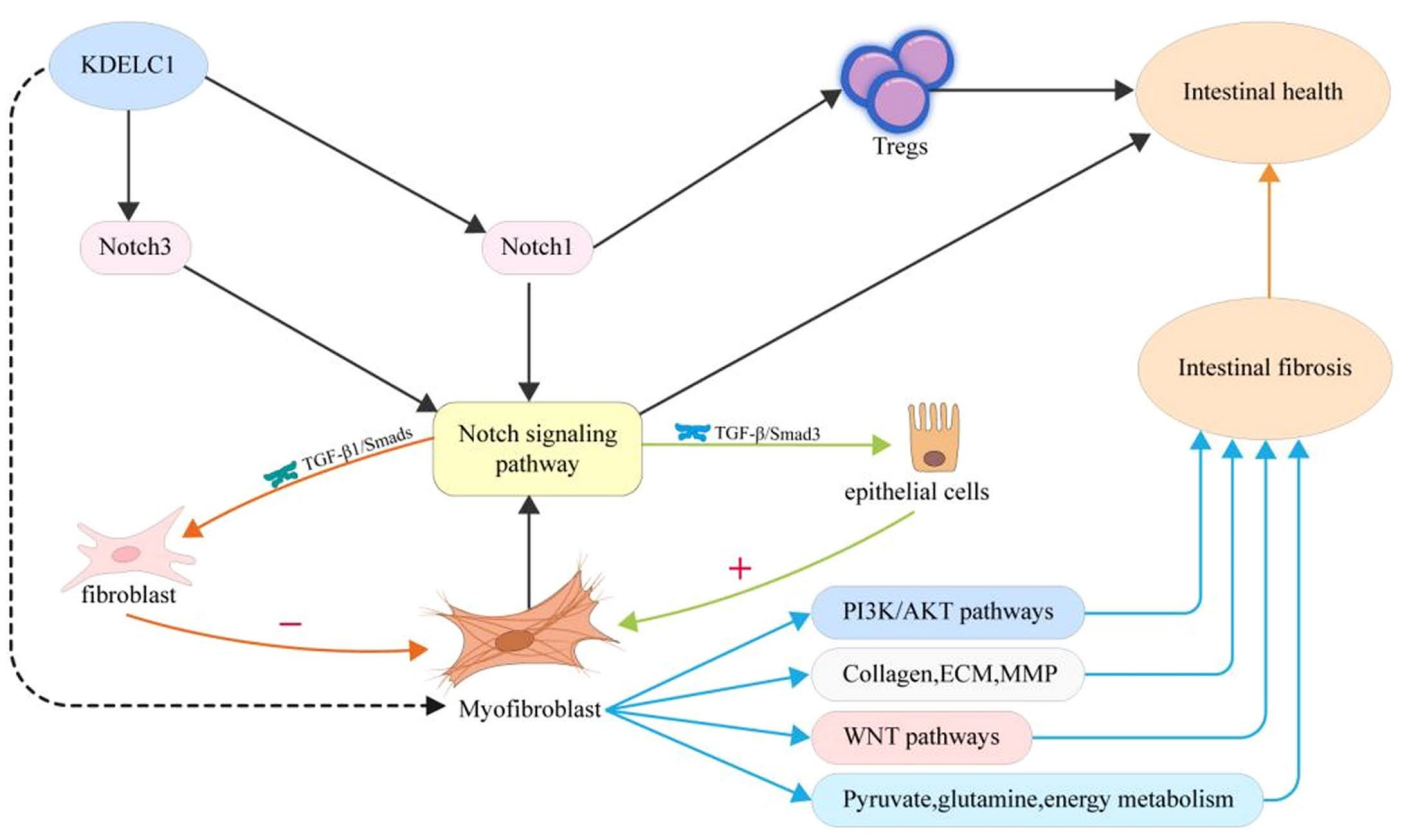
Proposed mechanism of myofibroblast activity, gene expression, and metabolism changes in patients with CD upon the treatment of UST.

In this study, we linked the key gene KDELC1 to MF and explored the different states of MF in function, metabolism, and cell communication under different expression conditions (Figure 8). We found that MF with high expression of KDELC1 promoted TGF-β, TNF-*α*, and NF-kB pathways. We divided MF into two groups according to the expression of the key gene KDELC1 and made a volcano map to show the genes with significant differences. Studies have shown that Myosin Heavy Chain 11(Myh11) is used to label pericytes and can be used to track the transformation of pericytes into myofibroblasts (30). CSF2, a kind of colony-stimulating factor (CSF), is closely correlated with M1 macrophages in the intestinal mucosa of patients with IBD, which regulates the function of macrophages (31, 32). CH25H, cholesterol 25-hydroxylase (CH25H), is closely associated with the expression of intestinal immune homeostasis and markers of fibrosis. Experiments showed that deficiency of CH25H reduced the degree of intestinal fibrosis in mice (33, 34). Numerous studies have confirmed that these pathways play an important role in the pathogenesis of CD and the progression of fibrosis (6, 35).

The exploration of metabolomics contributes to the study of pathophysiological mechanisms of diseases and the identification of diagnostic biomarkers. A recent study revealed that through the management of the diet of CD patients, sustained remission of CD was closely related to sustained changes in metabolism (36). Interestingly, our study suggests that KDELC1 may influence metabolic conditions by regulating MF. When KDELC1 is highly expressed, the metabolism of vitamin B6 and various amino acids is affected. Studies have found that the absorption of vitamin B6 in patients with CD is often decreased due to small intestine involvement and malnutrition. In addition, compared with healthy people, patients with CD have a significant reduction of vitamin B6 (37). A recent retrospective analysis showed that vitamin B6 abnormalities in CD patients were independently correlated with enteral and parenteral lesions and the use of immunosuppressants, and they also affected the structure of intestinal flora (38).

Previous experiments have confirmed that arginine can reduce the levels of mucosal permeability, oxidative stress, and pro-inflammatory cytokines (39). At the same time, it has been directly pointed out that the remission of CD is closely related to the significant increase in arginine levels (36). Through the analysis of metabolites in serum and stool of the healthy control group and CD patients, it was found that lipids, amino acids, and metabolites related to energy metabolism in CD patients had significant changes (40–43). Recent studies have confirmed that lipid-related metabolites can be used as new biomarkers for CD diagnosis (44). Based on the above analysis, it was found that, compared with healthy adults, lipid metabolism, amino acid metabolism, and energy-related metabolism were affected in patients with CD, which suggested that the change in metabolic status may be an important factor in the pathogenesis of CD.

The role of various cell types in the gut and the interactions between cells are crucial to understanding the mechanisms of intestinal fibrosis (45). Changes in the intestinal microenvironment may affect the recruitment or activation status of cells, which is also closely related to the development of intestinal inflammation and fibrosis (46). MF is a participant in maintaining intestinal homeostasis, and its activation and differentiation are considered to be key events in the process of fibrosis (47). Moreover, cytokines produced between various cells in chronic intestinal inflammation are important drivers of the occurrence and development of intestinal fibrosis. Therefore, we screened MF as the core cell population for subsequent analysis. Based on single-cell analysis, we found that the key gene KDELC1 may affect the communication and interaction between MF cells and other cells through the analysis of the cell-to-cell communication network. Analysis showed that the ligand-receptor pairs, including APP-CD74, CXCL12-CXCR4, and MIF-(CD74 + CXCR4), mediated the communication between IMF-related subtypes and B cells, T cells, macrophages, and endothelial cells. Previous studies have shown that CD99 is expressed on the surface of white blood cells, participates in the regulatory process of migration, and is positively correlated with the activity of CD (48). Studies have confirmed that MIF cytokines increase colon inflammation and play an important role in promoting the proliferation of intestinal epithelial cells and maintaining the integrity of the mucosal barrier by acting on CD74 receptors (49). In addition, MIF can also bind and activate CXCR2, CXCR4, and CXCR7 to regulate inflammation (50, 51). Therefore, it can be expected that regulating the expression level of KDELC1 and thereby affecting MF metabolism and cell–cell communication may be helpful for the clinical treatment of CD.

Despite the valuable insights gained from our data sets, several limitations to our study should be acknowledged. While we found that fibrosis and collagen I deposition in the narrowed intestine were significantly reduced after 1 year of UST treatment compared to baseline, no significant improvements in intestinal stenosis were observed through endoscopy. We speculate that this may be due to the relatively short duration of UST maintenance treatment, suggesting that longer observation periods may be necessary for further evaluation. Although the role of KDELC1 in CD remains underexplored, our data indicate its influence on the function of myofibroblast (MF) cells, at least in fibrotic tissues. Further investigation is required to fully understand the effects of KDELC1 on other cell types within the intestinal tissue. For instance, other researchers have reported evaluations of transmural healing, which is clinically relevant for studying intestinal fibrosis (52). In addition, our study’s small sample size limits the broader clinical relevance of our findings. A larger cohort or access to additional datasets are needed to validate and extend our conclusions.

## 5 Conclusion

In summary, we identified the functions and pathways affected by UST therapy in CD through bioinformatics methods, revealing that UST alleviates intestinal fibrosis by regulating MF metabolism and intercellular communication by targeting KDELC1. Our study provides new therapeutic targets for CD, which may have clinical value in the future.

## Data Availability

The datasets presented in this study can be found in online repositories. The names of the repository/repositories and accession number(s) can be found at: https://www.ncbi.nlm.nih.gov/, GSE112366.

## Footnotes

^1^ https://www.ncbi.nlm.nih.gov/geo/

^2^ http://geneontology.org/

^3^ https://ngdc.cncb.ac.cn/celltaxonomy/

^4^ https://github.com/neurorestore/Libra
